# Autoantibody Specificities in Myasthenia Gravis; Implications for Improved Diagnostics and Therapeutics

**DOI:** 10.3389/fimmu.2020.00212

**Published:** 2020-02-14

**Authors:** Konstantinos Lazaridis, Socrates J. Tzartos

**Affiliations:** ^1^Department of Immunology, Hellenic Pasteur Institute, Athens, Greece; ^2^Department of Neurobiology, Hellenic Pasteur Institute, Athens, Greece; ^3^Tzartos NeuroDiagnostics, Athens, Greece

**Keywords:** autoimmunity, myasthenia gravis, autoantibody, diagnosis, therapy, acetylcholine receptor, MuSK, LRP4

## Abstract

Myasthenia gravis (MG) is an autoimmune disease characterized by muscle weakness and fatiguability of skeletal muscles. It is an antibody-mediated disease, caused by autoantibodies targeting neuromuscular junction proteins. In the majority of patients (~85%) antibodies against the muscle acetylcholine receptor (AChR) are detected, while in 6% antibodies against the muscle-specific kinase (MuSK) are detected. In ~10% of MG patients no autoantibodies can be found with the classical diagnostics for AChR and MuSK antibodies (seronegative MG, SN-MG), making the improvement of methods for the detection of known autoantibodies or the discovery of novel antigenic targets imperative. Over the past years, using cell-based assays or improved highly sensitive immunoprecipitation assays, it has been possible to detect autoantibodies in previously SN-MG patients, including the identification of the low-density lipoprotein receptor-related protein 4 (LRP4) as a third MG autoantigen, as well as AChR and MuSK antibodies undetectable by conventional methods. Furthermore, antibodies against other extracellular or intracellular targets, such as titin, the ryanodine receptor, agrin, collagen Q, K_v_1.4 potassium channels and cortactin have been found in some MG patients, which can be useful biomarkers. In addition to the improvement of diagnosis, the identification of the patients' autoantibody specificity is important for their stratification into respective subgroups, which can differ in terms of clinical manifestations, prognosis and most importantly their response to therapies. The knowledge of the autoantibody profile of MG patients would allow for a therapeutic strategy tailored to their MG subgroup. This is becoming especially relevant as there is increasing progress toward the development of antigen-specific therapies, targeting only the specific autoantibodies or immune cells involved in the autoimmune response, such as antigen-specific immunoadsorption, which have shown promising results. We will herein review the advances made by us and others toward development of more sensitive detection methods and the identification of new antibody targets in MG, and discuss their significance in MG diagnosis and therapy. Overall, the development of novel autoantibody assays is aiding in the more accurate diagnosis and classification of MG patients, supporting the development of advanced therapeutics and ultimately the improvement of disease management and patient quality of life.

## Introduction

Myasthenia gravis (MG) is an autoimmune disease, characterized by muscle weakness and fatiguability of skeletal muscles (1, 2). MG is antibody-mediated, caused by autoantibodies targeting components of the neuromuscular junction (NMJ). Autoantibody binding causes impaired neuromuscular transmission, either by damage of the postsynaptic muscle membrane or by disruption of its normal organization.

**Figure 1:**
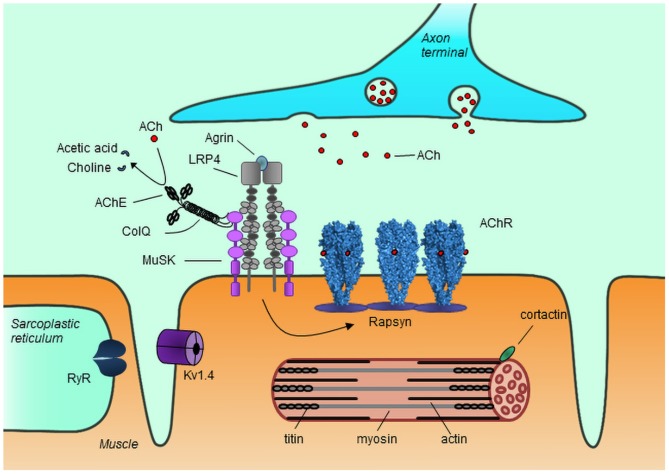
Schematic representation of the neuromuscular junction and myotube components. Agrin released from the nerve terminal binds to LRP4, which in turn binds to and activates MuSK, causing rapsyn-mediated AChR clustering. Acetylcholine (Ach) released from the nerve terminal binds to AChR causing opening of the receptor channel and triggering muscle contraction. Unbound acetylcholine in the synaptic cleft is broken down into choline and acetic acid by AChE, thus terminating its action. The antigenic targets for autoantibodies in MG known so far are depicted, though not all have been shown to be implicated in pathology. AChR, acetylcholine receptor; MuSK, muscle specific kinase; LRP4, low-density lipoprotein receptor-related protein 4; RyR, ryanodine receptor; ColQ, collagen Q; AChE, acetylcholinesterase; Kv1.4, voltage gated potassium channel 1.4.

The NMJ is responsible for transmission of the signal from the axon terminals of motor neurons to the muscle, rapidly translating neuron action potentials into muscle contraction. Acetylcholine released from the axon terminals binds to and activates the muscle acetylcholine receptors (AChRs), triggering opening of the receptor channel and depolarization of the muscle membrane. The AChRs are clustered at the NMJ resulting in localized high density of receptor clusters, which increases the efficiency of signal transmission. AChR clustering is driven by agrin, which upon release from the nerve terminals binds to low-density lipoprotein receptor-related protein 4 (LRP4), activating it to form a complex with muscle specific kinase (MuSK), thus causing the autophosphorylation and activation of MuSK. This results in a signaling cascade that promotes rapsyn-mediated AChR clustering at the NMJ (3, 4) (Figure 1).

MG is heterogeneous in terms of symptom presentation, with focal or generalized weakness, as well as in terms of pathophysiology, since different NMJ antigens can be targeted (5, 6). The symptoms usually initially manifest at the ocular muscles; in some patients they remain localized (ocular MG, OMG), while in the majority of patients the symptoms progress to other skeletal muscles within a couple of years (generalized MG, GMG). The disease presents with two peaks of incidence, below or above the age of 50, termed early-onset MG (EOMG) and late-onset MG (LOMG), respectively.

Although MG is a rare disease, with a prevalence of 150–300 per million population and an incidence of ~10 per million per year (7), it is considered a model antibody-mediated autoimmune disease, since in most cases the autoantibodies and target antigens are well-characterized. The majority of patients (~85%) have antibodies against the muscle AChR. Furthermore, antibodies against MuSK are found in approximately 6% of the patients, while relatively recently antibodies against LRP4 have been found in about 2% of MG patients. The pathogenicity of all these autoantibodies has been shown by the development of passive transfer experimental autoimmune MG (EAMG) when injected into laboratory animals and by the improvement of patients' symptoms following plasmapheresis (8–10). Some patients do not have detectable antibodies against any of these antigens, being referred to as seronegative (SNMG). Antibodies against various other extracellular or intracellular targets are found in several MG patients. Although the pathogenicity of these molecules is often uncertain or unlikely, they can still be highly informative disease biomarkers.

The detection of autoantibodies is crucial for MG diagnosis and for the differential diagnosis of many disorders with similar presentation. We will review the main autoantibodies found in MG, the advances toward development of increasingly sensitive detection methods and the identification of new antibody targets in MG. Furthermore, since the antigen targeted can dictate the response to treatment and novel advanced therapeutics aim to be antigen-specific, we will discuss their significance in therapy.

## MG Autoantibodies and Their Detection

### Antibodies Against the AChR

The autoantibodies in the majority of MG patients are directed against the muscle AChR of the NMJ. The muscle AChR is composed of five homologous subunits with a stoichiometry of α_2_β*γδ* in fetal or denervated muscles and α_2_β*δε* in adult muscles (11). Each subunit has a highly structured extracellular domain (ECD), four transmembrane domains and a partly structured intracellular domain. The autoantibodies target the ECDs of the AChR subunits and are very heterogeneous, since autoantibodies against all five subunits can be found in the same patient, including the γ subunit of the fetal AChR (12–15). Despite this, approximately half of the autoantibodies bind to the α subunit and especially the main immunogenic region (MIR), formed by overlapping epitopes located on the α1 subunit ECD, whose central core lies between amino acids 67–76, although other segments contribute as well (16–18). Furthermore, the autoantibodies against the α subunit are more pathogenic than those against the other subunits (10).

The AChR antibodies belong primarily to the IgG1 and IgG3 subclasses (19, 20). They can, therefore, activate complement at the postsynaptic membrane and thus cause AChR loss and destruction of its characteristic architecture, which is necessary for efficient signal transduction (21). Additionally, being bivalent, they can cross-link receptors leading to their endocytosis and destruction (antigenic modulation) (22). Finally, autoantibodies that bind close to the ligand binding site can directly interfere with receptor activation by acetylcholine (23).

Serological testing for the detection of AChR antibodies is often the first step for MG diagnosis, along with electrophysiological examination and assessment of response to acetylcholinesterase (AChE) inhibitors. The titer of AChR antibodies does not correlate with disease severity, although some evidence suggests that such a correlation emerges when the titer of only the MIR-directed, or the IgG1 subclass antibodies is considered (20, 24). In individual patients, on the other hand, the titer is associated with symptom severity and with response to therapy (25). Indeed, in a recent case study, gradually increasing AChR antibody titers were detected retrospectively up to 2 years before the onset of typical MG symptoms (26). Therefore, testing serial samples from the same patient attains added importance for monitoring their progress and guiding disease management. Additionally, the AChR antibody titer could provide information with respect to the risk of transient neonatal MG (TNMG), since it appears that TNMG is probable when the mother's titer is above 100 nM, but unlikely when it is below 10 nM (27).

The most widely used method for AChR antibody detection currently is the radioimmunoprecipitation assay (RIPA) (28). It is based on the indirect labeling of human AChR with ^125^I-α-bungarotoxin, which is a highly specific antagonist for the AChR (29). Sources of AChR can be human muscle or, more commonly, AChR-expressing cell lines, such as the CN21 cell line, which has been engineered to express both the fetal and adult types of the receptor (i.e., ε-expressing TE671 cells) (30). The AChR antibody RIPA has been the golden standard in MG diagnosis for many years due to its very high specificity (approximately 99%), as well as sensitivity, which is about 85% in the case of generalized MG and about 50% in ocular MG (31). In rare cases AChR antibodies can be found in patients with other autoimmune disorders or with thymoma without MG (32). The RIPA is also quantitative, allowing for detailed autoantibody titer determination, which is useful for monitoring individual patients.

A simple but much more sensitive RIPA than the classical one, has also been developed, which allows decreasing the cut-off for positivity from 0.5 to 0.6 nM AChR antibodies to <0.1 nM. It involves the use of 16 times larger serum volumes mixed with the usual amount of radiolabeled AChR followed by precipitation with the minimum required amount of semi-purified anti-human IgG antibody, to avoid increasing the background. By this approach, 20 of 81 tested SN-MG Chinese patients were found AChR antibody positive (33).

Enzyme-linked immunosorbent assay (ELISA) for AChR antibodies is also available (34), but it is not as sensitive as the standard RIPA (35) and consequently it has not gained as much traction in routine diagnosis as RIPA. Furthermore, radiological and ELISA assays have been developed to specifically detect modulating or blocking antibodies, but they marginally increased the sensitivity compared to the standard RIPA (36, 37). Another promising non-radioactive alternative to the RIPA is a fluorescence immunoprecipitation assay (FIPA), whereby the target antigen is labeled with fluorescence. This method has been shown to have relatively good overall sensitivity and specificity, but it is still not as good as the RIPA, and although it circumvents the hazards of radioactivity, it requires specialized equipment and expertise, making it difficult for routine diagnosis (38). Finally, an approach based on labeling of the recombinant AChR α subunit with *Renilla* luciferase and measuring the precipitated fluorescence by serum autoantibodies was able to detect AChR antibodies in 32% of MG patients (39). The low sensitivity could be in part due to the use of a fragment of the α subunit and further investigation is needed to assess its potential role in MG diagnosis.

Over the last few years, the application of cell-based assays (CBAs) has been gaining ground as previously undetected antibodies can be identified. The antigen is expressed in a suitable cell line, usually HEK293 cells, and the binding of autoantibodies is detected by a secondary fluorescently labeled antibody by microscopy. Specifically, in the case of AChR antibody CBAs the cells are also transfected with rapsyn in order to promote clustering of the receptors at the cell surface. This allows the detection of antibodies that will only bind to high density AChRs, resembling their organization at the NMJ, or of antibodies whose epitopes are destroyed or altered by the detergent solubilization of membranes for the isolation of AChR antigen. Initially, using this CBA 60% of previously SN-GMG and 50% of SN-OMG patients were found to have AChR antibodies (40, 41), though subsequent studies had varying results ranging between 4 and 38% of previously SNMG patients (33, 42–44). Routine diagnosis indicates that the overall fraction of SNMG sera positive for clustered AChR antibodies may be ~20% (45). The sensitivity of the assay is greater when both the adult and fetal form of the receptor are used (46). The CBA-detected antibodies were shown to belong to the same subclasses as the RIPA-detected antibodies and to potentiate complement depositions on the cell surface, indicative of a similar pathogenesis. However, patients with AChR antibodies detectable only by CBA seem to present with milder symptoms and better response to treatment (43).

Several studies have shown that the CBA can detect AChR antibodies which are not detectable by the classical diagnostics (38, 43, 47, 48). The CBA also has the advantage to be able to distinguish between antibodies against the fetal or adult form of the receptor (46). This becomes relevant in the diagnosis of cases of transient neonatal MG not associated with maternal MG, whereby the antibodies only recognize the fetal AChR leaving the adult AChR practically unaffected and the mother without signs of MG (13, 49, 50). On the other hand, in our own experience, the CBA lacks the quantitative resolution of the RIPA and thus cannot provide detailed titers for disease monitoring, while it often fails to detect autoantibodies in sera of very low but positive titer by the RIPA.

Finally, efforts are made toward the establishment of easy to perform instrument-free rapid assays, that could be used in non-specialized facilities (small clinics or neurologists' offices), since this could greatly reduce the time to diagnosis and improve patient management. To this end, we have developed a modified ELISA based on immobilization of AChR onto a solid support stick (immunostick), which has shown to have good specificity and sensitivity (99 and 91%, respectively) for AChR antibodies (51). Moreover, the immobilization of additional antigens in different zones on the immunostick could allow the simultaneous detection of more than one MG autoantibodies by this method. A similar approach based on blotting AChR preparations onto a nitrocellulose membrane, resulting in a dot-blot method, achieved the same sensitivity as the ELISA (52). Although such methods could be beneficial for MG diagnosis, they require further evaluation before clinical application.

### Antibodies Against MuSK

MuSK is a muscle membrane protein, which has an extracellular domain, a transmembrane helix domain and a cytoplasmic domain with tyrosine kinase activity. The extracellular domain includes three immunoglobulin-like regions and a cysteine-rich domain, also called Frizzled-like domain. The majority of MuSK antibodies bind to the Ig-like regions of the MuSK extracellular domain (53, 54). MuSK antibodies are detected in ~6% of all MG patients, or 40% among the AChR antibody negative patients. This ratio varies among countries with a lower prevalence in Northern Europe and higher toward the Mediterranean, probably owing to geographical and genetic differences (53, 55–59). In Japanese populations MuSK-MG seems to be less common with an overall prevalence of 2–3% (60). Until recently, detection of MuSK antibodies in AChR antibody positive patients was very rare (61, 62).

Unlike AChR antibodies, MuSK antibodies belong primarily to the IgG4 subclass, which do not activate complement and are largely functionally monovalent due to Fab arm exchange (63, 64). Their pathogenicity appears to stem from inhibition of interactions between MuSK and collagen Q or LRP4 via binding to the first Ig-like domain of MuSK and subsequent reduction of both agrin-induced and agrin-independent AChR clustering (65–67). The titer of MuSK antibodies appears to correlate with disease severity, both in individual patients and in the population (68, 69).

MuSK antibodies are routinely detected by RIPA using directly ^125^I-labeled MuSK (70). In an effort to increase the sensitivity of the RIPA, an alternative two-step method has been developed, whereby in the first step the MuSK antibodies, which may be at very low titers, are concentrated from large serum volumes by means of affinity chromatography, while the second step is effectively the standard RIPA (71). This approach allows the use of up to 50 times larger serum volumes, which would otherwise cause increased non-specific binding.

Commercially available ELISAs for the detection of MuSK antibodies are available but less commonly used. As a non-radioactive alternative to RIPA, FIPA seems very promising as the two assays have been shown to have the same sensitivity (38). Importantly, the FIPA could be performed so that both AChR and MuSK antibodies are measured simultaneously by labeling each antigen with a different fluorescent dye, thus potentially reducing the cost and time for diagnosis.

CBAs for MuSK antibodies have also been developed over the last years, which have detected MuSK antibodies in previously SNMG patient sera, including Asian populations (38, 40, 47, 72). Screening of 633 SNMG sera from 13 European countries revealed a prevalence of 5–22% for MuSK antibodies depending on the country (44). Interestingly, most of the detected MuSK antibodies in this study belonged to the IgM and not the IgG class. The CBA allowed the detection of MuSK antibodies in SN-OMG patients as well, which is not common with the classical assays (38, 44). Furthermore, using CBAs the percentage of sera positive for antibodies to more than one antigen has increased. In more detail, AChR antibody positive patients were also found positive for MuSK antibodies in 0.5–12.5% of the patients (44, 73). It is conceivable that some double positive patients were not identified in the past, since those found seropositive for AChR antibodies were not routinely tested for MuSK antibodies.

### Antibodies Against LRP4

LRP4 has a central role in synaptic development and maintenance. It is a transmembrane protein, containing several low-density lipoprotein domains. LRP4 acts as the muscle receptor for neural agrin, propagating the signal to MuSK for AChR clustering at the NMJ (74). LRP4 autoantibodies are detected in some MG patients. Inhibition of the LRP4-agrin interaction appears to be responsible, at least in part, for their pathogenicity (75–78). However, LRP4 antibodies belong mostly to the IgG1 subclass (75, 78), and they have been shown to cause *in vitro* complement-mediated cell lysis of C2C12 myotubes (78), so complement activation could also play a role in MG patients.

Initial reports varied significantly with respect to the prevalence of LRP4 antibodies, reported from 2 to 45%, possibly due to variations in the detection assays (ELISA, CBA or immunoprecipitation), the source of the antigen used (animal or human) and the populations examined (Japanese or Caucasian) (75–77). Indeed, studies in Chinese populations suggested that LRP4 antibodies are less frequent than in Western countries, as they were only found in 1–2.9% of SNMG and 0.8–1.7% of the total MG patients, while they were associated mostly with OMG (73, 79). We used CBA to perform a multinational study with samples from 635 patients without detectable AChR or MuSK antibodies. We found that 19% had LRP4 antibodies, corresponding to 2% of all MG patients, with considerable variability among the various countries (from 7% for Norway and Turkey to 33% for Poland) (80). Again, the use of these assays has revealed several cases of double positive patients; 15–20% of MuSK antibody positive and 7.5% of AChR antibody positive sera have been found positive for LRP4 antibodies as well (44, 80, 81). In addition to the NMJ, LRP4 is also present on motor neurons in the brain. Interestingly, LRP4 antibodies have also been detected in 10–23% of amyotrophic lateral sclerosis (ALS) patients and are thus not exclusively specific for MG (82, 83). Nevertheless, their detection can aid in the diagnosis of MG in parallel with the clinical presentation of the patients.

### Striational Antibodies

Striational antibodies were originally identified by staining of sarcomeres with patients' sera, which produced characteristic striational patterns. They are directed against several muscle fiber proteins, including titin, the ryanodine receptor (RyR), actin, myosin, tropomyosin, filamin, and others (84–86). Although all these proteins are important players in muscle contraction, their intracellular localization makes it unlikely for the respective autoantibodies to have a directly pathogenic role in MG. Nonetheless, titin and RyR antibodies are useful biomarkers and their detection can provide invaluable prognostic information.

Titin is a filamentous intracellular protein, the largest known so far, with a molecular weight between 3,000 and 4,200 kDa (87). Despite its size and repetitive nature, titin autoantibodies bind to a specific 30 kDa domain corresponding to 1% of titin's mass. This domain, known as MGT30, has been expressed as a recombinant titin fragment and is located near the A/I band junction (88). Titin antibodies are currently mostly detected in routine diagnosis by means of commercially available ELISA kits with the MGT30 domain. Overall, 20–40% of all AChR antibody positive patients have also been found positive for titin antibodies, with a marked age-related pattern; the prevalence is as low as 6% in EOMG and rises to 50–80% in non-thymomatous patients with LOMG (89–93). In EOMG patients titin antibodies are a strong indication for the presence of thymoma, as they are found in 50–95% of EOMG patients with thymoma, but only in few non-thymoma EOMG patients (86, 89, 90, 94–97). On the other hand, the presence of titin antibodies in all age groups appears to be related with more severe symptom manifestation (90, 95, 96, 98), although this relation has not been confirmed by all relevant studies (93). Using the aforementioned ELISA, titin antibodies have not been found in MG patients negative for AChR antibodies (93, 95, 98). More recently, we developed a RIPA method for the detection of titin antibodies using ^125^I-labeled MGT30, which has been used to screen a large cohort of European MG patients (99). The RIPA detected all the positive sera found by the ELISA, but it also detected titin antibodies in 13.4% of SNMG patients, as well as in patients with MuSK and LRP4 antibodies (14.6 and 16.4%, respectively). Interestingly, the titin antibody titers were higher in sera also positive for AChR antibodies. Low titer titin antibodies found in SNMG did not correlate with the presence of thymoma. This is in agreement with the finding that patients without AChR antibodies irrespective of age group were very unlikely to present with thymoma (97). The symptom severity was the same among the titin antibody positive and negative SNMG patients, suggesting that the detection of titin antibodies in SNMG is not prognostic for more severe disease, but they are a valuable biomarker for MG diagnosis. Recently, despite its intracellular localization, a “cytometric CBA” was developed for the detection of titin antibodies, based on incubation of stably titin-transfected HEK293 cell with serum samples and secondary labeled antibodies, followed by FACS analysis for quantitation of the results (100). This method showed increased sensitivity for titin antibodies compared to the ELISA when it was used to screen MG patients with myositis or myocarditis.

The RyR is a calcium channel located in the sarcoplasmic reticulum membrane and is involved in the excitation-contraction coupling mechanism by mediating Ca^2+^ release from the sarcolemma to the cytoplasm. RyR antibodies can be detected by western blot using crude sarcoplasmic reticulum or by ELISA using a fusion protein containing the main immunogenic domain of the RyR (101). The presence of RyR antibodies in patients has been found to differ between MG subgroups. Similar to titin antibodies, they are usually absent in EOMG, while they can be found in up to 40% of LOMG patients. Moreover, they are present in up to 75% of MG patients with thymoma (95, 102, 103). Overall, their presence has been correlated with more severe disease manifestation (104, 105).

### Antibodies Against Other Antigens

In addition to the aforementioned, several other antigenic targets have been reported in MG, although their pathogenicity, specificity for MG and diagnostic or prognostic value have not been fully characterized. These include the proteins agrin, Kv1.4 potassium channel, rapsyn, cortactin, acetylcholinesterase (AChE), collagen Q (ColQ) and collagen XIII.

Agrin is a proteoglycan secreted by the motor neuron, which then binds to muscle LRP4 and activates a signaling cascade resulting in AChR clustering. Antibodies against agrin have been detected in sera of MG patients ranging from 2 to 15% by ELISA or CBA (81, 106–108). Although most agrin antibody positive sera were also positive for AChR, MuSK or LRP4 antibodies, some were SNMG. This finding, together with the absence of detectable agrin antibodies among the samples from healthy controls or patients with other neurological diseases (such as multiple sclerosis, ALS, and neuromyelitis optica), support their diagnostic value as MG-specific autoantibodies. Importantly, patients with agrin antibodies presented with mild to severe symptoms and moderate response to treatment, thus their early detection could aid in disease management (81). Agrin antibodies appear to be pathogenic, since in *in vitro* studies they were capable of inhibiting MuSK activation by agrin and AChR clustering (108), while immunization of mice with neural, but not muscle, agrin induced MG-like symptoms (109).

The voltage gated potassium channel α-subunit Kv1.4 is expressed mainly in neurons of the central nervous system, where they control presynaptic release of acetylcholine. They are also found in skeletal and heart muscles. Studies of antibodies against Kv1.4 in Japanese MG populations, revealed that they were present in 11–18% of the patients and their presence was correlated with severe symptoms, myasthenic crises, and thymoma (110–112). Furthermore, it was found that 11–27% of Kv1.4 antibody positive Japanese MG patients also suffered from or had clinically suspected myocarditis, the clinical onset of which was always preceded by the detection of Kv1.4 antibodies, while 36–60% presented with abnormal ECG findings. On the other hand, investigation of a Caucasian population revealed the same Kv1.4 antibody prevalence among MG patients (17%), but their presence was associated with female LOMG patients with mild symptoms, in many cases purely OMG (113). It appears therefore, that only in Japanese populations, the Kv1.4 antibodies are an important biomarker indicating increased risk of myocarditis or cardiac dysfunction among MG patients. However, their detection is difficult as it involves the immunoprecipitation of ^35^S-labeled cell extracts by patient sera followed by SDS-PAGE electrophoresis: the presence of a 70 kDa Kv1.4 band in rabdomyosarcoma extracts but not in leukemic cell extracts is considered a positive finding (110). The application of a cytometric CBA could prove a useful alternative, as it has recently been successfully used to detect Kv1.4 antibodies with similar efficiency to the RIPA (100).

Rapsyn is an intracellular muscle protein, which acts as a scaffold, linking the intracellular domain of the AChR with the cytoskeleton and thus mediating receptor clustering at the NMJ (114). Rapsyn antibodies are found in up to 15% of MG patients, including SNMG (115). However, the fact that no associations have been identified with disease severity or MG subgroups, while they are also detected in several other autoimmune diseases, such as systemic lupus erythematosus (116), diminishes their diagnostic potential.

Cortactin is a cytoplasmic protein involved in actin assembly and MuSK-induced AChR clustering at the NMJ. Cortactin antibodies were first identified in patient sera using a human protein array (117). Further analyses using ELISA and western blot for confirmation of the results, detected cortactin antibodies in up to 23.7% of SNMG samples and 9.5% of seropositive MG, suggesting that they can be valuable in SNMG diagnosis and prognostic of mild disease (117–119). However, cortactin antibodies have also been found in up to 12.5% of patients with other autoimmune diseases and 5.2% of healthy controls, while they have been described as myositis-associated, since they are found in 7.6–26% of patients with polymyositis, dermatomyositis and immune-mediated necrotizing myopathy (117, 120). Therefore, their relevance for MG diagnosis and contribution to pathogenesis still requires further investigation.

AChE is an enzyme localized at the synaptic cleft of the NMJ, where it catalyzes the breakdown of acetylcholine, thus terminating its action on AChRs. Antibodies against AChE have been reported in 5-50% of MG patients (121–123). No significant correlation was observed with sex, age of onset, or thymic pathology, while they were not MG-specific, as they were also found in several patients with other autoimmune diseases.

ColQ is found in the extracellular matrix at the NMJ, probably via interactions with MuSK, where it mediates the anchoring of AChE (124). Recently, antibodies against ColQ were found in a fraction of MG patients (3%) using CBA, including some SNMG patients, although they were also present in a similar fraction of the control cohort used in the study (125). The pathogenicity of ColQ antibodies has not been assessed so far. Therefore, their diagnostic value and potential pathogenic role remains to be elucidated.

Collagen XIII antibodies have been detected in 7.1% of AChR antibody positive MG patient and 15.8% SNMG sera screened (126). No discernible differences were seen among patients with and without collagen XIII antibodies in terms of symptom manifestation. Moreover, these antibodies are not MG specific and have been also associated with Grave's ophthalmopathy (127).

**Figure 2:**
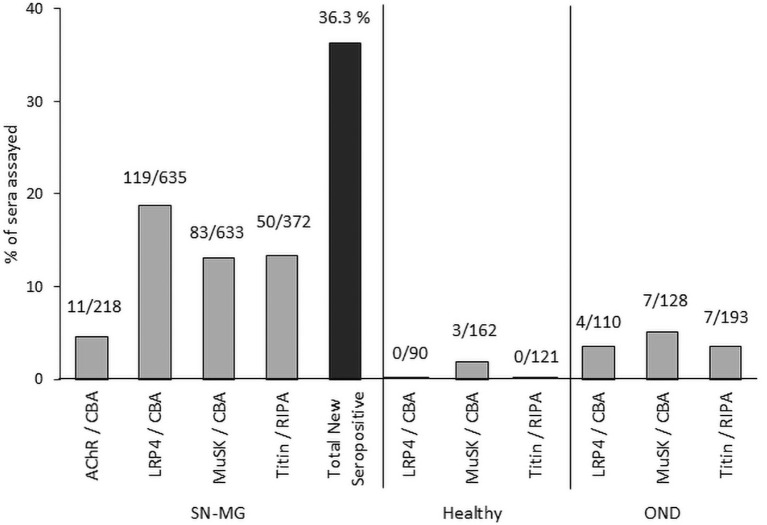
Detection of autoantibodies in SNMG by novel assays. We have used CBA and RIPA for screening a large number of MG patients without detectable autoantibodies by the classical assays, as well as several control samples from healthy individuals or patients with other neuroimmune diseases (OND), from 10 to 13 different European countries (44, 80, 99). The numbers above the bars indicate the number of positive samples and the total tested with each assay. The cumulative percentage (black bar) of new positives among the SNMG samples that were positive in more than one assays were taken into account, so as to avoid overestimation of the total new seropositive patients.

The above observations with respect to the different MG autoantibodies are summarized in Table 1, while Figure 2 shows the percentage of previously seronegative MG patients in which autoantibodies are found using some of the novel detection assays described, in a European cohort.

**Table 1:** Summary of autoantibody prevalence, usual detection method and major clinical associations where known^*^.

**Autoantigen**	**Detection method**	**% of MG patients**	**% of dSN-MG patients**	**Other diseases**	**Clinical associations**	**Representative references**
AChR	RIPA	80–85%	N.A.	Rare	Thymic abnormalities, thymoma	Several references, reviewed in Gilhus et al. (6)
Clustered AChR	CBA	N.T.	~20% (4–60%)	N.T.	Milder symptoms than AChR+ MG, thymic abnormalities	(40, 45)
MuSK	RIPA	~6% (2–3% in Japanese)	N.A.	Rare	Bulbar symptoms common, no thymic abnormalities	(53, 56, 60)
MuSK	CBA	N.T.	13%	5%	Milder symptoms	(44)
LRP4	CBA	~2%	~19%	3.6% (10–23% in ALS)	Milder symptoms than AChR+ MG, no thymoma	(80) (83)
Titin	ELISA	20–30% (90% in thymoma EOMG	0–3%)	Some	Correlation with thymoma in AChR+ EOMG	(86, 90, 94, 128)
Titin	RIPA	~41%	13.4%	0–3.6%	No correlation with thymoma	(99)
RyR	ELISA	~ 14% in LOMG (75% in thymoma MG)	N.T.	N.T.	Correlation with thymoma in AChR+ MG	(95, 103, 104)
Agrin	ELISA/CBA	2–15%	0–50%	13.8% in ALS	Mild to severe symptoms, moderate response to treatment	(83, 106)
Kv1.4	IP and SDS-PAGE	10–20%	0%	0%	Japanese: Severe symptoms, myasthenic crises, thymoma, cardiac involvement Caucasian: Mild symptoms in LOMG	(110, 112, 113)
Rapsyn	Immunoblots	11%	17%	10% OND 78% SLE	Not known associations	(115, 116)
Cortactin	ELISA, WB	5–10%	~20%	12.5%	Not known associations	(117, 118)
ColQ	CBA	3%	3.4%	5%	Not known associations	(125)

* *Some studies on potential antigens with small cohort sizes and non-MG-specific findings are not included in the table*.

*N.T., not tested or not extensively tested; N.A., not applicable; SLE, systemic lupus erythematosus*.

## Relevance for Therapy

The determination of the autoantibody specificity, in addition to its diagnostic value, is also very important for correct management of MG patients. Firstly, the detection of serum autoantibodies, especially in the case of AChR and MuSK, can provide a practically certain diagnosis for MG, allowing the initiation of appropriate treatment. Moreover, monitoring of the antibody titer can be very useful in following disease progression and response to therapy.

Importantly, the therapeutic regime can differ between the MG subgroups. Patients with MuSK antibodies tend to have more severe symptoms and generalized weakness (129), whereas treatment withdrawal in these patients can often lead to disease exacerbation. In addition, MuSK-MG patients can present with adverse effects when treated with pyridostigmine, an AChE inhibitor commonly used as a first-line treatment for MG, while there is little evidence to support the usefulness of thymectomy in these patients (130). On the other hand, they usually greatly benefit from plasma exchange (PLEX) (131), and they have a very good response to the administration of rituximab, possibly more pronounced than the other MG subgroups (132, 133). AChR antibody positive patients who also have titin or RyR antibodies tend to have more severe disease, while in the case of EOMG they are indicative of thymoma (93). The benefit of thymectomy is questionable in patients with SNMG, MuSK-MG and LRP4-MG since they usually lack the typical thymus pathology seen in AChR-MG (134). Especially in the case of Japanese patients, the presence of Kv1.4 antibodies has been associated with cardiac dysfunction and severe complications, so they should be monitored accordingly. It is, therefore, important to be able to diagnose the patients, not only based on clinical and electrophysiological examination, but also serologically. The detection of the autoantigen targeted in each patient is crucial to adopt the best treatment options.

The most common treatment strategies for MG currently include the use of AChE inhibitors, immunosuppressive agents, thymectomy, intravenous immunoglobulin (IVIG) and plasmapheresis (2, 135). These, however, are largely non-specific and thus may be accompanied by a variety of side effects, especially given the often life-long immunosuppressive treatment required. Novel therapies should aim to be antigen-specific, i.e., target the specific autoimmune components of the immune system, which are mostly well-known in MG. For the application of such tailor-made therapies the characterization of the patients' autoantibody specificities by serological tests is crucial.

One approach would be the selective removal of only the pathogenic autoantibodies (antigen-specific immunoadsorption). The method is similar to plasmapheresis, but in this case the plasma is passed through a suitable matrix, such as sepharose-immobilized autoantigens, to which the autoantibodies bind, while the rest of the plasma, free of autoantibodies, is returned to the patient (136, 137). Several studies have shown the efficiency of the approach, with significant fractions of the autoantibodies being removed from AChR and MuSK antibody positive MG patient sera *in vitro*, or from laboratory animals with EAMG *ex vivo* (54, 138). In the *ex vivo* studies immunoadsorption was shown to lead to significant amelioration of the symptoms within a few treatment sessions, while no adverse effects were seen (139, 140). No similar studies have been performed so far with LRP4 autoantibodies. Further tests are needed before clinical application of this approach, which should provide a solution when an immediate relief from MG symptoms is required (e.g., myasthenic crises, preoperatively) or for patients refractory to other treatments (132).

A different approach, aiming at treating the underlying pathology of MG, is to induce antigen-specific immunosuppression or immune tolerance for the targeted antigen, depending on the antibody specificities detected in each patient. To this end, several studies have shown that mucosal administration of AChR domains can lead to prevention or amelioration of ongoing EAMG (141–143). Prevention of EAMG was likewise achieved when T cell dominant peptides of the AChR ECDs were given orally or nasally (144, 145). Interestingly, when T cell dominant epitopes were administered in the form of subcutaneous immunization in the presence of adjuvant, a beneficial effect was also observed (146). A similar strategy relied on the use of peptide constructs incorporating only the intracellular sequences from all the AChR subunits (147). Although oral or nasal administration of the intracellular polypeptides was able to prevent and, in some cases, treat ongoing EAMG, the effect was greater when treatment was given as subcutaneous vaccination (148, 149). The therapeutic effect in the studies using ECD domains or their peptides was mediated by a shift of Treg cell responses from Th1 to Th2, a reduction in IFN-γ, IL-2, and IL-10 production levels and a switch of autoantibody subclass from IgG2b to IgG1. On the other hand, administration of the AChR intracellular domains relied on diverting the immunological response away from producing ECD-targeting pathogenic antibodies, toward epitopes of the intracellular domains, and possibly causing apoptosis of AChR-specific plasma cells. In our experience and several published studies, the therapeutic efficacy appears to depend on the conformation of the administered antigens and the route of administration (150–152). Given the advances in the heterologous expression of the AChR domains in various systems (15, 153), the elucidation of the precise mechanism and the specific immune cells involved would allow the design of increasingly targeted and specific therapeutic tools.

## Conclusions

Serological tests for the detection of autoantibodies are central in MG diagnosis. MG pathogenesis, its clinical presentation and the response of patients to therapy vary depending on the pattern of autoantibodies detected. In fact, the autoantibody specificity pattern is often more informative for symptom severity than the autoantibody titer.

The very high specificity of AChR and MuSK antibodies for MG, which are the predominant antigens, and their successful use so far justify their use as early diagnostics in cases of clinically suspected MG. Despite the requirement for radioactivity and, consequently, specialized laboratories, RIPAs provide very sensitive results with reliable antibody titer information, and are thus proposed as the initial tests for routine MG diagnosis. Due to increases in antibody concentration during disease progression and/or epitope spreading, repeated tests should be performed when a suspected MG patient is initially seronegative.

A percentage of MG patients remain seronegative, but since autoimmune MG is most likely mediated by autoantibodies in all patients, SN-MG patients probably have autoantibodies against yet unidentified target proteins, low affinity or low concentration antibodies against the known antigens, requiring different diagnostic tests. More sensitive assays for known antigens or the discovery and validation of novel autoantibodies is thus necessary. To this end, considerable efforts have been made toward improvement of the tests; CBA for MuSK, LRP4 and clustered AChRs, RIPA for titin and two-step RIPA for AChR and MuSK have contributed significantly toward the reduction of the percentage of SNMG patients and are thus increasingly used in routine diagnosis for the detection of MG autoantibodies (33, 42, 154, 155). Furthermore, the discovery of antibodies against several other antigens whose diagnostic relevance remains to be fully assessed, should aid in the elimination of MG patients without a classical serological marker for diagnosis. Interestingly, several CBAs have recently been developed for intracellular MG antigens. However, due to the inability of CBAs to provide titer information and the lack of available commercial kits they are proposed as second line tests in patients that remain seronegative by the standard RIPAs. The use of cytometric CBAs could be a useful alternative, should they prove to reliably and specifically produce quantitative results (100). Efforts are also being made to develop diagnostic assays that can be easily performed in non-specialized and equipment-free settings to further decrease the time from sample collection to diagnosis (51). Assays based on CBAs or immunosticks could be adopted to simultaneously detect more than one antigen. The use of several antigen-expressing cells immobilized on different dots on a slide, or immunosticks onto which antigens have been immobilized in different zones could allow testing for all the major MG autoantibodies. Such an approach would decrease the time and the cost for diagnosis. The expansion of proteomic methods could result in the screening for binding to several MG and other autoimmune disease antigens aiding not only in faster diagnosis for MG but also in the differential diagnosis of related disorders. However, such approaches are still far from being used in the routine diagnosis for MG.

The sensitive and accurate detection of autoantibodies in MG patients' sera also has important implications for therapy, supporting the development of advanced therapeutics. Personalized treatment for MG patients would be highly beneficial, and it would rely on characterization of MG pathogenic antibody specificities. Antigen-specific therapies, such as immunoadsorption or induction of immunological tolerance against AChR, MuSK, and LRP4 should be the focus of efforts for future treatments (156). Many studies have shown the proof of concept for various such approaches, but their therapeutic efficacy and mechanism of action needs to be fully elucidated with vigorous preclinical and clinical trials, before they can progress into clinical practice.

## Author Contributions

KL and ST researched the bibliography for the review, made substantial contributions to the content, and reviewed and edited the manuscript. KL wrote the first draft.

### Conflict of Interest

ST has shares in the research and diagnostic laboratory Tzartos NeuroDiagnostics. The remaining author declares that the research was conducted in the absence of any commercial or financial relationships that could be construed as a potential conflict of interest.
